# Statistical modeling of STR capillary electrophoresis signal

**DOI:** 10.1186/s12859-019-3074-0

**Published:** 2019-12-02

**Authors:** Slim Karkar, Lauren E. Alfonse, Catherine M. Grgicak, Desmond S. Lun

**Affiliations:** 1 Center for Computational and Integrative Biology, Rutgers University, Camden, 08102 NJ USA; 2 Biomedical Forensic Sciences Program, Boston University School of Medicine, Boston, 02118 MA USA; 3 Department of Chemistry, Rutgers University, Camden, 08102 NJ USA; 4 Department of Computer Science, Rutgers University, Camden, 08102 NJ USA; 5 Department of Plant Biology, Rutgers University, New Brunswick, 08901 NJ USA

**Keywords:** DNA degradation, Capillary electrophoresis, STR genotyping, Stochastic analysis and modelling

## Abstract

**Background:**

In order to isolate an individual’s genotype from a sample of biological material, most laboratories use PCR and Capillary Electrophoresis (CE) to construct a genetic profile based on polymorphic loci known as Short Tandem Repeats (STRs). The resulting profile consists of CE signal which contains information about the length and number of STR units amplified. For samples collected from the environment, interpretation of the signal can be challenging given that information regarding the quality and quantity of the DNA is often limited. The signal can be further compounded by the presence of noise and PCR artifacts such as stutter which can mask or mimic biological alleles. Because manual interpretation methods cannot comprehensively account for such nuances, it would be valuable to develop a signal model that can effectively characterize the various components of STR signal independent of a priori knowledge of the quantity or quality of DNA.

**Results:**

First, we seek to mathematically characterize the quality of the profile by measuring changes in the signal with respect to amplicon size. Next, we examine the noise, allele, and stutter components of the signal and develop distinct models for each. Using cross-validation and model selection, we identify a model that can be effectively utilized for downstream interpretation. Finally, we show an implementation of the model in NOCIt, a software system that calculates the *a posteriori* probability distribution on the number of contributors.

**Conclusion:**

The model was selected using a large, diverse set of DNA samples obtained from 144 different laboratory conditions; with DNA amounts ranging from a single copy of DNA to hundreds of copies, and the quality of the profiles ranging from pristine to highly degraded. Implemented in NOCIt, the model enables a probabilisitc approach to estimating the number of contributors to complex, environmental samples.

## Background

Biological material collected from the environment is routinely used as a substrate for DNA testing with applications including human identification in forensic science, ancient DNA analysis in anthropology, the evaluation of transplant success in medicine, the identification of modified crops in the food industry, and fishery and wildlife survey in ecology [1–3]. Since the 1980s, laboratories conducting human identity testing have targeted hypervariable microsatellite regions of DNA known as Short Tandem Repeats (STRs) which consist of variably sized repetitive sequences. The general workflow consists of isolating DNA from cellular material, then amplifying a set of sequences using the polymerase chain reaction (PCR). Commonly used human identification assays currently amplify 13 to 24 loci. Each of the loci is composed of repeating units of up to 7 base pairs, and amplicons typically range in length from less than 100 to greater than 300 base pairs [4–6].

**Capillary Electrophoresis and DNA Sequencing** After PCR, amplified STRs are typically identified via Capillary Electrophoresis (CE) and, sometimes, next-generation sequencing (NGS). Although NGS is well-established in innumerable fields, its use in human identity testing remains limited by the relatively slow pace at which standards and guidelines are issued by the FBI [7] and the Scientific Working Group on DNA Analysis Methods (SWGDAM, [8]). With the first set of guidelines concerning the interpretation of STR data obtained from NGS systems only recently published in April 2019, CE is likely to persist as a go-to method for achieving fine-grain separation of STR amplicons, with modern platforms facilitating automated analysis of hundreds of samples in one day.

**Analysis of environmental samples** CE quantifies the amount of STR amplicons of a given size in Relative Fluorescence Units (RFU). Traditionally, analysis of the RFU signal begins with applying a threshold to separate interpretive signal from noise. Next, the genetic profile(s) of the contributor(s) are deduced using a combination of presence/absence rules [9]. This method has been shown to result in inaccurate interpretation of forensic samples that contain (i) a low mass of DNA, (ii) a mixture of DNA from several individuals, or (iii) damaged or degraded DNA [8, 10, 11]. Alternative methods that employ complex, continuous models of the signal have been developed to facilitate the interpretation of challenging forensic samples; these models can be used within a likelihood ratio (LR) framework to evaluate the strength of the evidence [12–14].

***Model of DNA degradation***

**Fig. 1:**
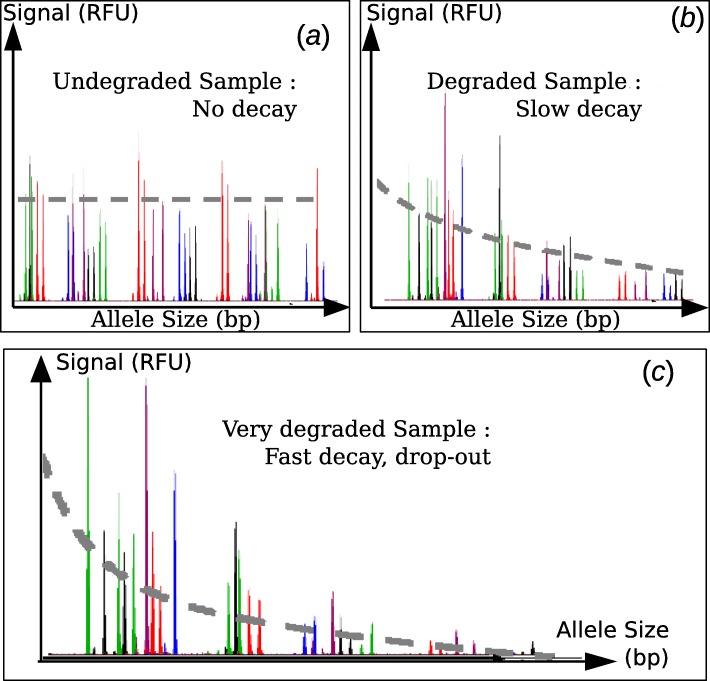
The effect of DNA degradation on CE-STR profiles for (**a**) an untreated sample exhibiting no decay, (**b**) a sample degraded with 24 mU rDNase I exhibiting moderate decay, and (**c**) a sample exposed to UV radiation for 105 min exhibiting both fast decay and drop-out of high molecular weight alleles. All profiles were obtained from the same whole blood donor, amplified with the GlobalFiler™ PCR Amplification Kit at 0.25 ng, and injected for 15 s on the Applied Biosystems 3500

Regardless of the application, when biological material is obtained from an uncontrolled environment, the DNA present in the sample is often degraded or damaged through exposure to microorganisms, UV radiation, or acidic conditions. In addition, compounds that are collected with the biological material may co-extract with the DNA and inhibit PCR. In forensic samples, the major processes resulting in DNA degradation include strand cleavage from enzymatic degradation (e.g. DNase I in [15]), hydrolytic and oxidative reactions, as well as UV exposure [16]. In environmental samples such as biological stains of unknown origin in forensic cases, the combination of these different processes preferentially affects alleles of higher molecular weight. Therefore, degraded samples typically exhibit low peaks or even drop-out for alleles of larger size (See Fig. 1 and [17–19]).

**Degradation as a random process** Degraded, damaged, or inhibited profiles typically show an exponential decay [20] in which RFU signal decreases as the molecular weight of the allele increases (Fig. 1). This decay is known to be consistent with a Poisson process [21, 22]. As such, we model the degradation of the source DNA fragment (target) as a random process with a rate *λ* expressed in degradation events per base pair (bp). According to the Poisson model, the probability that a target of length *s* is not degraded and, hence, available for amplification is *p*(*s*)=*e*^−*λs*^. The rate *λ* reflects the level of degradation of the sample; for example, in the case of degradation through UV radiation, it reflects both the intensity and time of exposure. If there are *n* copies of a target of size *s* before degradation, the expected number of copies available for amplification (i.e. after degradation occurred) is *n*. *e*^−*λs*^.

Models of the PCR reaction [23, 24] show that we can expect a proportional relationship between the number of copies initially available for amplification and the number of product amplicons. Since the expected intensity of the CE signal (the peak height at the allele position) is proportional to the number of product amplicons, we have:

$${H(s) \ =\ A\ .\ e^{-\lambda s}}$$

where the constant *A* models the number of amplicons and their quantification through fluorescence (in RFU per amplicon). Previous studies [25–29] show that an affine, proportional peak height is a reasonable model. This model is consistent with the interpretation that the probability distribution of peak heights at allelic (true) positions is composed of a combination of amplicon signal and baseline noise.

### Probabilistic modeling of CE-STR profiles

A CE profile consists of peaks observed at multiple loci (typically 13 to 24). Peaks are characterized by their height, measured in RFU. When a peak corresponds to the genotype of a known contributor to the sample, it is referred to as an allelic or true peak. Stutter peaks, which result from strand slippage during PCR, typically present as one STR repeat unit larger or smaller than the biological allele [30]. Our model accounts for both forward and reverse stutter peaks (*n*+1 and *n*−1 stutter), and all other peaks are classified as (background) noise.

Occasionally, the allele of a contributor does not give rise to a peak: this phenomenon, called drop-out, is characterized by its frequency. In a similar fashion, we characterize the frequency at which stutter and noise peaks fail to arise and refer to these instances as stutter and noise drop-out, respectively. In total, the model has 8 components: (1) true (allelic) peaks, (2) forward stutter peaks, (3) reverse stutter peaks, (4) noise peaks, and their drop-out counterparts: (5) true drop-out, (6) forward stutter drop-out, (7) reverse stutter drop-out, and (8) noise drop-out. Peak height variables are modeled using Gaussian random variables, and drop-out events are modeled as Bernoulli random variables.

#### Model selection

Among several alternative models for peak heights and drop-out models, we seek to identify the best model and the correct explanatory variables. To compare models, the basic strategy is to choose the model with the lowest out-of-sample prediction error. The log likelihoods $\mathcal {L}_{h} ({f})$ and $\mathcal {L}_{DO} ({f})$ of a model *f* are used as a measure of the prediction error. The prediction error and the log likelihood are inversely related; thus, we define the prediction error $\mathcal {L}^{*}(f)=-\mathcal {L}(f)/N$, where *N* is the number of test samples.

To estimate the out-of-sample prediction error for a set of models $\mathcal {F}=\{(f_{1}, \cdots, f_{l}\}$, we employ *k*-fold cross-validation (with *k*=10) [31] separately on each dataset.

For each model $ f \in \mathcal {F} $ we compute ${\mu }_{\mathcal {L}}(f)$ and ${\sigma }_{\mathcal {L}}(f) $ the mean and (unbiased) standard deviation of 6×*k* out-of-sample prediction error estimates (one for each fold of cross-validation for each of the 6 datasets). For stutter peaks and stutter drop-out, log-likelihoods for reverse and forward datasets are pooled together, leading to 12×*k* out-of-sample prediction error estimates.

To select a model, we use a common model selection rule [31]: we select the most parsimonious model *f*^∗^ (i.e., the model with the lowest number of free parameters) such that ${\mu }_{\mathcal {L}}(f^{*}) < \left ({\mu }_{\mathcal {L}}(f_{min}) + {\sigma }_{\mathcal {L}}(f_{min}) \right)$, where $\left ({\mu }_{\mathcal {L}}(f_{min}), {\sigma }_{\mathcal {L}}(f_{min}) \right)$ are the mean and the standard deviation of the model with minimum error prediction ${\mu }_{\mathcal {L}}(f_{min})$. In the event that there is more than one model of the same dimension satisfying ${\mu }_{\mathcal {L}}(f^{*}) < \left ({\mu }_{\mathcal {L}}(f_{min}) + {\sigma }_{\mathcal {L}}(f_{min}) \right)$, we use other criteria for selection, such as the biological and chemical rationale of the model, as well as its computational cost.

## Results

The model described herein, compatible with the above referenced continuous LR framework, is distinct from the previous model in several aspects. First, we used over 1200 single source empirically derived multiplex STR profiles from pristine, degraded or damaged DNA, or inhibited PCR processes to develop the model [32]. Second, the models were developed to describe the chemistry of the PCR - namely the distribution of the number of amplicons as gamma distributions. While most of the methods account for drop-out probability, all of them rely on the application of an Analytical Threshold (AT) to remove noise peaks. Here, we utilize a combination of Gaussian models which consider explicitly the probability of drop-out and frequency of noise peaks. Among several alternative models, we seek to identify the best model and the correct explanatory variables. This family of models, which are both tractable and computationally sound, can describe multi-contributor samples (i.e., signal arising from more than one individual) [26, 33].

***True peak model***

We consider five models for a peak arising from a true (heterozygote) allele, denoted TP1 to TP5. TP1 to TP4 all have four free parameters *θ*=(*a*,*b*,*c*,*d*). TP5 has five free parameters. All five models are fitted using Maximum Likelihood Estimator *L*_*h*_ and use affine functions as in Eq. 1 (see Methods - Model Components).

**DNA template model TP1.** This model (similar to the one in [26]) uses *x*=*c*_*DNA*_, the template (DNA concentration or amount) of the sample. The template *c*_*DNA*_ is a measurement obtained using qPCR (see Methods). Note that for a given *c*_*DNA*_, the expected peak height will be the same for all alleles, regardless of locus and dye colors. This model accounts for undegraded samples.

**Degradation Index model TP2.** We set $x= c_{DNA}. e^{-\lambda.(s_{i}-s_{1})} \phantom {\dot {i}\!}$, where *s*_*i*_ is the length of peak (the allele) *i* in base pairs, *s*_1_ the length of the smallest autosomal target sequence, and *λ* is the degradation rate estimated from the DI value *q* for the sample obtained from qPCR (see Methods).

**Undegraded amplitude model TP3.** For each dye color *c*, we use an undegraded amplitude model, for all alleles of size *s*_*i*_ at all loci of dye color *c* : *x*_*i*_=*A*_*c*_, estimated by fixing parameter *B*_*c*_=0 in the quantification (see Decayed Amplitude in Methods).

**Decayed amplitude model TP4.** Given (*A*_*c*_,*B*_*c*_) the set of quantification parameters of the sample for dye color *c* (see Methods), we use the decayed amplitude $x_{i}=A_{c}\ .\ e^{B_{c}. s_{i}}\phantom {\dot {i}\!}$.

**Decayed amplitude model TP5.** We define another decayed amplitude model where we introduce an extra free parameter *j* to account for locus-specific degradation : $x_{i}=A_{c}\ .\ e^{B_{c}. s_{i} /j}\phantom {\dot {i}\!}$.

**Fig. 2:**
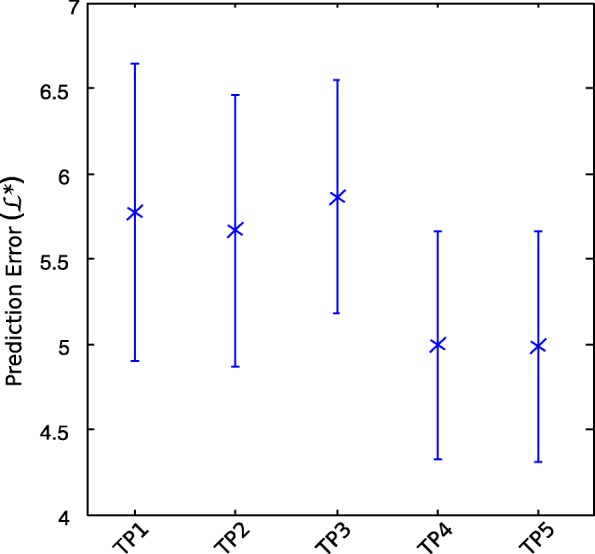
Out-of-sample prediction error for allelic (true) peak models. Markers indicate mean values, and bars extend to ± one standard deviation. k=10 out-of-sample prediction errors per dataset were estimated using cross validation. Over the 6 datasets, we obtained 60 values for each model, with: (TP1) DNA template model; (TP2) Degradation Index model; (TP3) Undegraded, amplitude model; (TP4) and (TP5): Decayed amplitude models with *n*=4 and *n*=5 free parameters

The out-of-sample prediction error measurements obtained by cross-validation for the five true peak models we considered are shown in Fig. 2. The decayed amplitude model with four parameters was selected as it outperformed other models of similar or lower complexity.

***Stutter models***

We investigate three families of models for stutter peaks. SP1 and SP2 model peak heights using Maximum Likelihood Estimator *L*_*h*_ and affine functions with four free parameters (see Methods), SR1 uses stutter ratio with five free parameters, SPE1 to SPE3 use nested models with up to nine free parameters.

**Affine Models for Peak Heights SP1 and SP2.** Affine Fit Parent Peak Height model SP1 uses *x*_*i*_=*PPh*_*i*_, the height of the parent allele peak. Affine Fit Decayed Amplitude model SP2 uses $x_{i}= A_{c}\ .\ e^{B_{c}. s_{i}}\phantom {\dot {i}\!}$, the decayed amplitude for an allele of size *s*_*i*_.

**Affine stutter ratio model SR1.** A common approach to characterize stutter peaks is to model the stutter ratio *r*_*i*_=*h*_*i*_/*PPh*_*i*_, where *h*_*i*_ is the height of the stutter peak and *PPh*_*i*_ is the height of the parent allele peak. In the case of an undegraded sample, this ratio has been shown to decrease exponentially with the amount of DNA template [26]. The stutter ratio’s random variable in SR1 follows a gaussian distribution : ${\cal {N}}(u_{r},v_{r})\left \{ \begin {array}{l} u_{r}(x_{i}) = a. e^{(-b.x)} +c \\ v_{r}(x_{i}) = j. e^{(-b.x)} +k \end {array} \right. ; \theta =(a,b,c,j,k) $.

**Exponential model with Parent Peak Height SPE1, SPE2 and SPE3.** In cases in which there are a low number of DNA copies, (i.e., low template or degraded samples), the stutter peak, its parent peak, or both peaks may be in the range of baseline noise; as such, the stutter ratio can be very high and can exceed 1. Some models circumvent this scenario by defining the stutter ratio using the sum of the stutter and parent peak heights [14, 34]. In a similar fashion, we defined a series of models with *x*_*i*_=*PPh*_*i*_, the height of the parent allele peak, defined as follows:

SPE1 : $ \left \{ \begin {array}{l} u(x)=x.(a.e^{-b.x+c}) \\ v(x)=x.(j.e^{-b.x+k}) \end {array} \right., \theta =(a,b,c,j,k) $ ;

SPE2: $ \left \{ \begin {array}{l} u(x)=x.(a.e^{-b.x+c})+m \\ v(x)=x.(j.e^{-b.x+k})+n \end {array} \right. ; \theta =(a,b,c,j,k,m,n) $ ;

SPE3: $ \left \{ \begin {array}{l} u(x)=x.(a.e^{-b.x+c})+m \\ v(x)=x.(j.e^{-l.x+k})+n \end {array} \right. ; \theta =(a,b,c,j,k,l,m,n) $

**Fig. 3:**
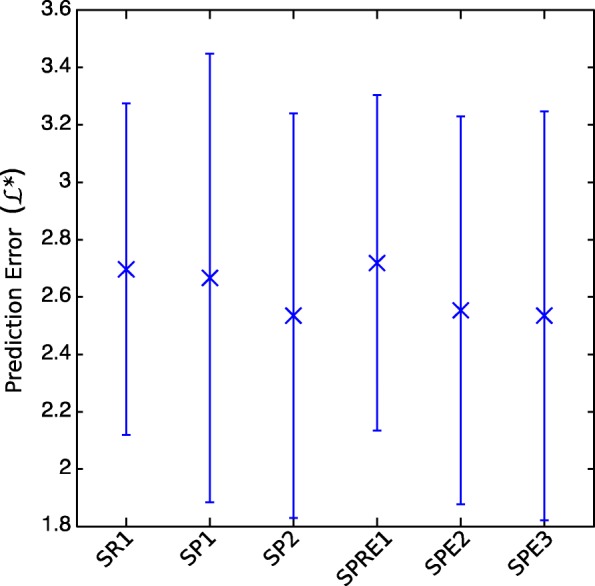
Out-of-sample prediction error for stutter peaks, reverse and forward. Markers indicate mean values, and bars extend to ± one standard deviation. k=10 out-of-sample prediction errors per dataset were estimated using cross validation. Over the12 datasets (6 reverse stutter, 6 forward stutter), we obtained 120 values for each model, with: (SR1) Stutter ratio model with parent peak height; (SP1) Peak height model with decayed amplitude; (SP2) Peak height model with parent peak height model; (SPE1 to SPE3) Exponential parent peak height models with *n*=5 to *n*=8 free parameters

Models were selected for reverse and forward stutter to maintain consistency. Models using stutter ratio and decayed amplitude (see Fig. 3) appeared the least accurate. All other studied models performed similarly over the datasets, as shown in Fig. 3. Ultimately, the affine peak height model using parent peak height was selected since it achieved the best performance with low complexity.

***Noise models***

Noise has been shown to be proportional to the DNA amount in [26]. Recently, [35] showed that log-normal modeling of noise peak heights performs better than a normal model, though the normal distribution cannot be excluded as a model. Our noise model encompasses several artifacts commonly excluded in noise studies [11, 36–38] such as N + 2 and N - 2 (double-back) stutters, and half repeat unit stutters that are present, for example, at loci SE33 and D1S1656. We investigate three models (NP1 to NP3) that all use Maximum Likelihood Estimator *L*_*h*_ and affine functions.

**Undegraded Model NP1.** This model of dimension four uses *x*_*i*_=*A*_*c*_, the amplitude of the signal at dye color *c*.

**Decayed Amplitude Models NP2 and NP3.** Model NP2 has four free parameters and uses $x_{i}= A_{c}\ .\ e^{B_{c}. s_{i}}\phantom {\dot {i}\!}$, the decayed amplitude for allele of size *s*_*i*_.

Model NP3 has five free parameters, four from NP2 plus an extra parameter *j* : $x_{i}= A_{c}\ .\ e^{B_{c}. s_{i}/j}\phantom {\dot {i}\!}$ to account for locus-specific degradation.

**Fig. 4:**
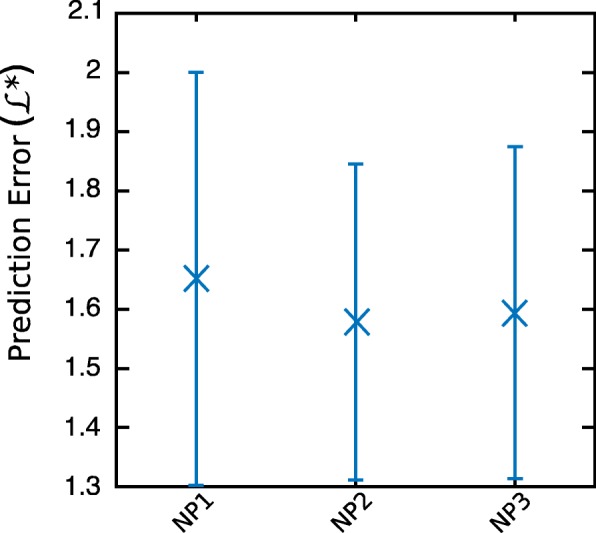
Out-of-sample prediction error for noise peak models. Markers indicate mean values, and bars extend to ± one standard deviation. k=10 out-of-sample prediction errors per dataset were estimated using cross validation. Over the 6 datasets, we obtained 60 values for each model, with: (NP1) Undegraded amplitude model; (NP2) Decayed amplitude model, n=4; (NP3) Decayed amplitude model, n=5

Decayed amplitude appears to be the best explanatory variable, particularly at higher injection times (Fig. 4). The most parsimonious decayed amplitude model was selected.

***Drop-out models***

We investigate drop-out using three families of models, Exponential Regression, Logistic Regression, and constant frequency. All drop-out models are fitted by maximizing *L*_*DO*_ (see Methods).

**Allelic drop-out models TDO1 and TDO2.** These drop-out models both have two free parameters *θ*=(*a*,*b*) and use $x_{i}= A_{c}\ .\ e^{B_{c}. s_{i}}\phantom {\dot {i}\!}$, the decayed amplitude for allele of size *s*_*i*_. Exponential Regression model TDO1 uses exponential function : *p*(*DO*)=*a*.*e*^−*bx*^. Decayed Logistic Regression model TDO2 uses logistic function : $p(DO)=1-\frac {1}{1+e^{-b(x-a)}}$.

**Stutter drop-out models SDO1 and SDO2.** Stutter dropout models use the Exponential Regression function *p*(*DO*)=*a*.*e*^−*bx*^. Parent Peak Height model SDO1 uses *x*_*i*_=*PPhi*, the height of the parent allele peak. Decayed Amplitude model SDO2 uses $x_{i}= A_{c}\ .\ e^{B_{c}. s_{i}}\phantom {\dot {i}\!}$, the decayed amplitude for allele of size *s*_*i*_.

**Noise drop-out models NDO1 and NDO2.** Decayed Amplitude model NDO1 uses the decayed amplitude and the Exponential Regression function. Constant frequency model NDO2 uses a constant function *p*(*DO*)=*a*.

**Fig. 5:**
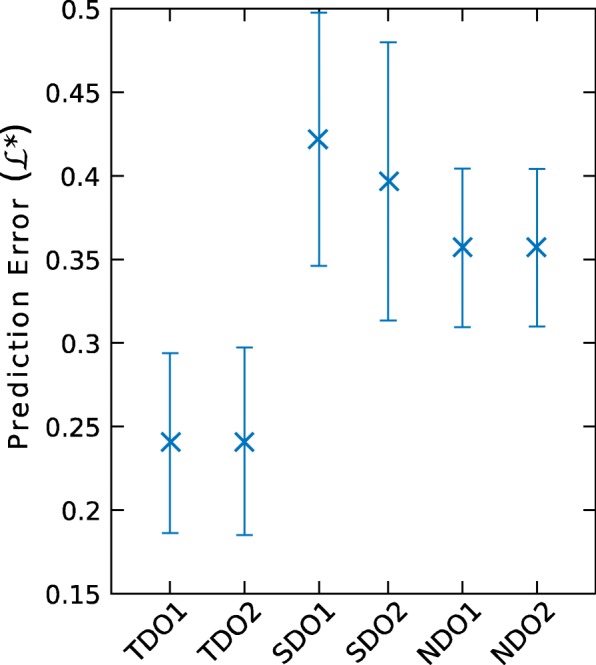
Out-of-sample prediction error for drop-out components. Markers indicate mean values, and bars extend to ± one standard deviation. k=10 out-of-sample prediction errors per dataset were estimated using cross validation. From left to right: Allelic drop-out (6 datasets) with: Allele drop-out models (TDO1) exponential, decayed amplitude model and (TDO2) logistic, decayed amplitude model; Stutter drop-out, exponential models (6 reverse, 6 forward datasets) with (SDO1) using Parent Peak Height and (SDO2) using decayed amplitude; Noise drop-out models (6 datatsets) with (NDO1) using constant model and (NDO2) using exponential model with decayed amplitude

For allelic drop-out (TDO1 and TDO2 on Fig. 5), the Exponential and Logistic models provided similar results. Since exponential regression is consistent with previous studies [26], the exponential form was used for all other drop-out components.

For stutter drop-out (SDO1 and SDO2 on Fig. 5), both models performed similarly. The model using parent peak height as the explanatory variable was selected, however, because it provides consistency with the explanatory variable for the stutter peak model.

For noise drop-out (NDO1 and NDO2 on Fig. 5), both models exhibited similar prediction error. The constant model was selected because it is more parsimonious.

### Software implementation

**Fig. 6:**
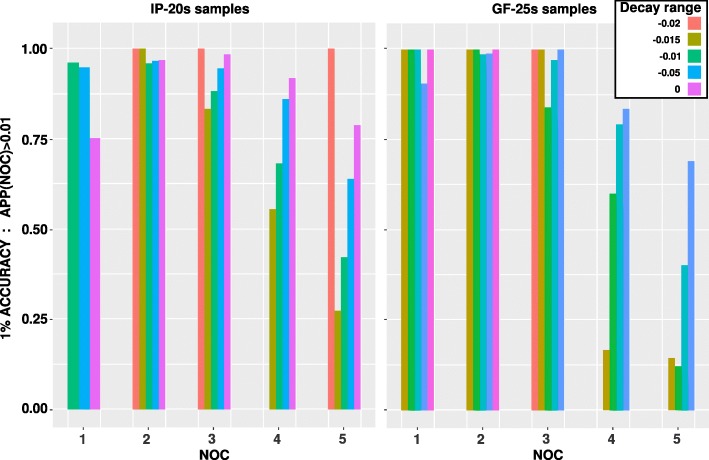
Accuracy of the *a posteriori* probability (APP) of the Number of Contributor (NOC) using the selected model components. For *n*=1 to *n*=*N*_*max*_=5, the APP is produced using an extended version of NOCIt [26] that accounts for differential degradation. **1% accuracy statistic** is the frequency, over 819 Identifiler Plus samples (left panel) and 826 GlobalFiler samples (right panel), for which NOCIt produced an APP >0.01 for the true number of contributor(s) in the sample. For each sample, the *B* value of the decayed amplitude model was used to define 5 categories of degradation reported as *Decay range*

Data and selected models of the components (see Table 1) are implemented in the NOCIt/CEESIt software suite available on the PROVEDIt website [39]. Briefly, NOCIt is a statistical software that performs a probabilistic evaluation of the number of contributors of a DNA sample. It computes the distribution of the *a posteriori* probability *P*(*N*=*n*|*E*),*n*=1,…*N*_*max*_ for an evidence sample *E* of having *n*=*N* contributors (see Methods and [26]). We extended the algorithm to account for differential degradation rates, implemented the selected models and conducted a study using over 800 DNA mixtures of 1 to 5 contributors from the PROVEDIt database [32]. The profiles contain DNA from 1 to 5 contributors; the contributor mixture ratios and template DNA amounts vary; and the profiles range in quality from pristine to severly comprised. Fig. 6 presents statistics on the *a posteriori* probability (APP) calculated by NOCIt for *n*=1 to *n*=*N*_*max*_=5 contributors. Accuracy of the APP is computed as the frequency at which the APP of the actual number of contributor (NOC) is higher than 1% (*P*(*N*=*NOC*|*E*)>0.01). Performance of the model is excellent for the less ambiguous, less degraded samples, and exhibits an expected decline for the more complex, compromised samples.

**Table 1:** Models for Peaks and Drop-out components

Component	Model	Input	Likelihood function
True peak	$\mathcal {N}(\mu,\sigma) ; \left \{\begin {array}{l} \mu =u(x) = a.x +b \\ \sigma =v(x) = c.x +d \end {array}\right.$	${x_{i}=A_{c}.e^{B_{c}.s_{i}}}\phantom {\dot {i}\!}$	$\mathcal {L}_{h}$
Noise peak			
Forward stutter		*x*_*i*_=*PPH*_*i*_	
Reverse stutter			
True peak D.O.	*p*(*x*)=*a*.*e*^*b*.*x*^	$\phantom {\dot {i}\!}{x_{i}=A_{c}.e^{B_{c}.s_{i}}}$	$\mathcal {L}_{do}$
Reverse stutter D.O.		*x*_*i*_=*PPH*_*i*_	
Forward stutter D.O.			
Noise peak D.O.	*p*(*x*)=*a*	*a*=*f*(*h*_*i*_)	

For each model component, at each locus, we indicate the probability distribution, its analytical form, and the model input *x*_*i*_, namely Decayed Amplitude for peaks in allelic and noise position, and *PPH*_*i*_ (parent peak height) for peaks in reverse and forward stutter position. Peak models follow a normal density, and the frequencies of drop-out are modeled using an exponential decay. Noise drop-out parameter *a* is independent of the observed sample. D.O. denotes drop-out

## Discussion

Contrary to models described elsewhere [13, 14, 34, 40], we separate the modeling of peak heights from the modeling of drop-out: in short, we aim to characterize the observed peaks rather than model the distribution of amplicons from individual genotypes. The stutter model we propose reflects the same approach.

We can examine the models we obtain to understand the characteristics of CE-STR profiles from the parameters of the model. We can then compare parameters values between loci of the same or different datasets. For example, the frequency of noise peaks, commonly referred to as drop-in, can be evaluated. In the GlobalFiler™ datasets, increasing the injection time from 5 to 25 seconds did not drastically affect the drop-in rate, with a typical median increase of 1.7% (maximum of 4%, minimum of 0.5%). The amelogenin locus exhibits different behavior, with a decrease in noise of 5% (see noise drop-out rate in Additional file 2: Table S2).

Another informative quantity is the expected amplitude of the baseline noise relative to the overall signal, which can be evaluated with the *a* parameter of the Noise model *u*(*x*)=*a*.*x*+*b*. This parameter can be roughly interpreted as the expected proportion of the total signal that is, on average, attributable to a single noise peak. These values (see Additional file 1: Table S1) were not significantly affected by the injection time. In addition, the value of second parameter, *b*, exhibited a small increase in the range of 0.1 to 1 RFU, suggesting that our noise model is robust and applicable to a variety of template DNA masses and instrument settings.

For many applications, evaluation of the drop-out rate is critical. However, such estimation is not straightforward since it is conditioned on both the template DNA mass and level of degradation. Using our model on single-source samples, one can evaluate the expected drop-out rate based on the signal amplitude rather than the template mass and degradation index. For example, using the Identifiler Plus kit with 10-second injection, the drop-out model parameters of locus D5S818 (Additional file 2: Table S2) are (*a*=0.684; *b*=0.0139); thus, to ensure a drop-out rate lower than 1%, a sample should exhibit total signal amplitude of at least 130 RFU (Details available at [41]). For a given signal amplitude, our model estimates the true (allelic) peak height. Given the parameters of the true (allelic peak) model of, for example, the locus D5S818 (see Additional file 1: Table S1), for a single source heterozygote sample, a signal amplitude of 130 RFU yields allelic, heterozygous peaks of height 63 RFU (or 126 RFU for homozygous individuals) on average. In a similar fashion, when signal exhibits peaks of 40 RFU from a heterozygous, single-source, one could expect a drop-out rate of 5%, and 10% for signal that contains peaks of 30 RFU.

## Conclusion

We propose a continuous, probabilistic model for CE-STR signal where we utilize the observed amplitude of the signal to model the DNA amount and level of degradation. Using a large amount of data, we evaluated several models for each component of the signal and selected the model that provides the best out-of-sample prediction error. Further development of this approach could extend to categorical data such as SNPs or micro-haplotypes. Next-generation sequencing data could also be investigated by modeling the number of reads, assuming that the flow cell is not saturated.

## Methods

### Samples and datasets

#### Extraction and generation of condition-dependent DNA samples

Single-source whole blood samples from a total of fifty donors were diluted to 1:10, 1:100, and 1:1000 in TE buffer and subjected to various protocols to generate untreated or compromised DNA, as described below. The number of donor cell lines treated with each protocol is summarized in Table 2. Generally, UV-damaged samples were extracted using the EZ1®DNA Investigator Kit on the EZ1®Advanced (Qiagen) following the manufacturer’s recommended protocols for Pretreatment for Various Casework and Reference Samples and DNA Purification (Large-Volume Protocol) [42]. All other sample types were extracted in 50 *μ*L aliquots using the QIAamp®DNA Investigator Kit (Qiagen) following the manufacturer’s recommended protocol for Isolation of Total DNA from Small Volumes of Blood or Saliva [43]. The elution volume was 50 *μ*L for both extraction methods.

**Table 2:** Summary of the different protocols utilized to generate extracts of differing condition. For each protocol, three levels were generated such that the extracts generally became more compromised as the level increased (i.e., due to increasing enzyme concentration, increasing incubation time, increasing sonication cycle number, etc.)

	Number of whole blood donors (n) for each condition			
	level			
Condition	I	II	III	N/A
**Untreated**				n=50
(mU)	6	12	24	
**rDNase I**	*n*=35	*n*=35	*n*=35	
(min)	15	30	45	
**Fragmentase**®	*n*=15	*n*=15	*n*=15	
(cycles)	2	10	30	
**Sonication**	*n*=14	*n*=14	*n*=14	
(min)	15	60	120	
**UV Damage**	*n*=22	*n*=22	*n*=22	
(*μ*L)	15	22	35	
**Humic Acid**	*n*=22	*n*=22	*n*=22	

The number of donors from which DNA extracts were obtained and subjected to the various protocols is indicated

##### (i)

Untreated samples were generated by extracting aliquots of each whole blood dilution as described above. These extracts were not subjected to any conditions intended to induce inefficiencies in amplification.

##### (ii)

rDNase I-degraded samples were produced using the DNA-free™Kit (Life Technologies). Three levels of degradation were generated by digesting extracts with 6, 12, and 24 mU rDNase I. The digestion parameters followed the manufacturer’s recommended protocol with a ten-minute incubation at 37C; the reaction was subsequently halted by proprietary enzyme inactivation [44].

##### (iii)

Fragmentase®-degraded samples were produced by extracting 50 *μ*L aliquots of each whole blood dilution using the QIAamp®DNA Investigator Kit and a modified elution volume of 37 *μ*L deionized water. Three levels of degradation were created using the NEBNext®dsDNA Fragmentase®Kit (New England Biolabs) by incubating extracts with the Fragmentase enzyme cocktail for 15, 30, and 45 min. The digestion parameters followed the manufacturer’s recommended protocol [45], and the reactions were halted by the addition of 10 *μ*L 0.5 M EDTA. To remove EDTA, all extracts subsequently underwent a second extraction following the manufacturer’s recommended protocol [43].

##### (iv)

Sonicated samples were generated by diluting extracts to a total volume of 200 *μ*L with TE buffer. The extracts were sonicated using the Fisher Scientific™Model 50 Sonic Dismembrator at 25% amplitude for two, ten, and thirty sonication cycles, where one cycle was defined as 30s sonication on followed by 30s sonication off.

##### (v)

UV-damaged samples were created by spotting 100 *μ*L aliquots of each whole blood dilution onto glass microscope slides and allowing the stains to air dry for 75 min. The stains were subsequently irradiated using the QIAgility®UV lamp for 15, 60, and 120 min. All stains were collected using the double swab method using cotton swabs moistened with deionized water [46]. Swabs were air dried overnight, then extracted as described above.

##### (vi)

Humic Acid-inhibited extracts were generated by combining 50 *μ*L aliquots of each whole blood dilution with 50 *μ*L Buffer ATL, 10 *μ*L Proteinase K, and 100 *μ*L Buffer AL (containing cRNA) [43]. These solutions were vortexed, incubated at 50^∘^C for 10 min, then briefly centrifuged. Three volumes (15, 22, and 35 *μ*L) of 2 mg/mL humic acid solution (Sigma Aldrich) were added to the cell lysate solutions which were subsequently incubated at room temperature for two hours, vortexing every 30 min to mix. After incubation, the extraction protocol was resumed to completion.

#### Quantification, amplification, capillary electrophoresis and analysis.

All extracts were quantified using Quantifiler®Trio DNA Quantification Kit (Applied Biosystems) on the Applied Biosystems®7500 using the manufacturer’s recommended thermalcycling protocol and an external calibration curve [47]. The concentration of the small autosomal target was used to calculate the appropriate volume of extract to amplify given the desired template mass. Extracts were amplified on the GeneAmp®PCR Amplification System 9700 using 9600 emulation mode with a gold sample block using the GlobalFiler®PCR Amplification Kit (Applied Biosystems) (29 cycles) following the manufacturer’s recommended protocol at the following target masses: 0.5, 0.25, 0.125, 0.063, 0.031, 0.016, and 0.008 ng [48]. Extracts were also amplified using the Identifiler®Plus PCR Amplification Kit (Applied Biosystems) (28 cycles) following the manufacturer’s recommended protocol (28 cycles) using the same thermalcycler and template masses specified above [49]. Positive and negative amplification controls were processed in tandem. Where necessary, dilutions were prepared in TE buffer. GlobalFiler®amplicons were injected for 5, 15, and 25 s at 1.2 kV on the Applied Biosystems®3500 Genetic Analyzer, and Identifiler®Plus amplicons were injected for 5, 10, and 20 s at 3 kV on the Applied Biosystems®3130 Genetic Analyzer. CE profiles were analyzed with GeneMapper®ID-X v1.4 at an analytical threshold of 1 RFU. The genotype table for each sample was exported from GeneMapper®as a CSV file containing the allele, size, and height for all peaks. Table 3 present a synthesis of peak calling. Artifacts in the profile, such as pull-up and complex pull-up, were filtered using NOCIt. The pull-up height ratio and size range were set to 6% and ±0.6 base pairs, respectively. The complex pull-up height ratio, sister height ratio, and size range were set to 6%, 50%, and ±0.3 base pairs, respectively.

**Table 3:** The number of peaks and drop-out peaks observed for each model component in the Identifiler™ Plus (IP) 5, 10 and 20 second and GlobalFiler™ (GF) 5, 15 and 25 second datasets

Dataset	Model component	# peaks	# drop-out peaks
IP	Allele	39,939	6329
5 second	Reverse	14,429	18,840
	Forward	4985	28,284
	Noise	53,577	461,466
IP	Allele	40,110	5,154
10 second	Reverse	17,857	14,603
	Forward	6478	25,982
	Noise	62,698	438,092
IP	Allele	39,318	4934
20 second	Reverse	20,092	11,709
	Forward	7817	23,984
	Noise	69,199	421,334
IP	Allele	53,175	10,807
5 second	Reverse	4942	43,855
	Forward	17,366	31,431
	Noise	79,436	1,005,765
IP	Allele	54,988	7320
15 second	Reverse	25,851	21,586
	Forward	8145	39,292
	Noise	90,603	962,560
IP	Allele	56,754	7186
20 second	Reverse	30,571	18,092
	Forward	10,587	38,076
	Noise	98,704	980,775

For each single source profile, peaks were categorized according to the known donor genotype as allele, reverse stutter, forward stutter or noise. When no peak was observed, the position was considered drop-out

### Characterization of degradation in DNA samples

**qPCR Degradation Index as a measurement of degradation** One way to evaluate the amount of degradation of a DNA sample is to estimate the ratio of the number of copies of two target sequences of differing length [20]. To this end, the **Degradation Index**, measured using realtime PCR (qPCR), has been proposed [50]. The Degradation Index is described as: *q*=*s*_1_/*s*_2_ where *s*_1_ and *s*_2_ are autosomal target sequences of 80 and 214 bp, respectively. It can be shown that this value is related to the degradation rate *λ* by the equation log(*q*)=−*δ*.*λ* where *δ*=*s*_2_−*s*_1_.

**CE signal-based characterization of the sample: Decayed amplitude** In the case of controlled, single-source samples, we expect the total signal at a given locus to be mainly driven by the total number of amplicons produced at that locus, which is proportional to the number of copies initially available for amplification. For degraded samples, that amount will follow an exponential decrease that depends on the size of the alleles. We argue that the evolution of the total signal across loci labeled with the same fluorescent dye is related to the sample degradation rate *λ*.

For each dye color *c*, we compute the decayed amplitude function ${f_{c} (s) =A_{c}\ .\ e^{B_{c} s}}\phantom {\dot {i}\!}$, where *A*_*c*_ is the expected signal amplitude, for color *c*, without degradation, and *B*_*c*_ is the decay factor, which reflects the degradation of the sample for color *c*. We define the amplitude of the signal for a given locus as the sum of all observed peaks (*h*_1_,⋯,*h*_*n*_) at the locus *l* : $H_{l}=\sum ^{n}_{1} h_{i}$. For a set of *N* loci (*l*_1_,⋯,*l*_*N*_) at a given dye color (usually 3≤*N*≤5), we have a set of *N* amplitudes (*H*_1_,⋯,*H*_*N*_). At a locus *l*, for *n* observed peaks of height at position of alleles of size (*s*_1_,⋯,*s*_*n*_) we define the weighted average size $\bar {s}_{i}$ of the alleles at the locus by :

$$\bar{s}_{l} =\frac{\sum^{n}_{1} h_{i}.s_{i}}{\sum^{n}_{1} h_{i}}$$

If the CE profile for a particular dye color presents at least two loci *l*,*m* for which we can compute ${\bar {s}_{l},\bar {s}_{m} }$, then an exponential regression curve of the form $f_{c} (s) =A_{c}\ .\ e^{B_{c} s}\phantom {\dot {i}\!}$ has a unique solution *A*_*c*_,*B*_*c*_. Thus, we define the **Decayed Amplitude**, for an allele of size *s*_*i*_ as $ x_{i}=A_{c}\ .\ e^{B_{c}. s_{i}}\phantom {\dot {i}\!}$. Note that if the CE instrument has the same sensitivity for all dyes, one can use loci from different dye colors for this computation. Such a characterization has two major features: (*i*) it does not require a separate measurement of the DNA amount (i.e., quantitation via qPCR) and (*ii*) it does not require prior knowledge of the alleles that are present in the sample (i.e., the contributor genotype). These two features enable characterization of the degradation of a sample regardless of its DNA template mass or allelic content.

### Model components

#### Peaks

The heights of true (allelic) peaks, stutter peaks (forward and reverse), and noise peaks are modeled as Gaussian distributions (*μ*,*σ*) with mean and standard deviation *μ*=*u*(*x*);*σ*=*v*(*x*), where *u* and *v* are functions of a given peak-dependent explanatory variable *x* (also referenced as input). As an example, the affine functions used in [26] is:

1 $$ \cal{N}(\mu, \sigma) \left\{ \begin{array}{l} \mu= u(x) = a.x +b \\ \sigma =v(x) = c.x +d \end{array} \right.  $$

where *θ*=(*a*,*b*,*c*,*d*) is the set of parameters for the model, which is estimated from data.

Single-source calibration data allow us to classify each observed peak as one of the four types: **true peak, reverse stutter, forward stutter, or noise**. Consider a sequence of *n* peaks of a specific type, of peak heights {*h*_1_,⋯,*h*_*n*_}. We estimate the set of parameters for a model using the Maximum Likelihood estimator *Θ*_*ML*_=arg max*θ*(*L*_*h*_), where

2 $$ {L_{h}=-{ \sum_{i = 1}^{n} \left({\log \left(v(x_{i}) \right) + { \frac { \left(h_{i} - u(x_{i}) \right)^{2}} {v(x_{i})^{2}}} } \right) }}.  $$

For peaks caused by stutter, we also develop models using the stutter ratio, which for peak *i* is ${r}_{i} = \frac {h_{i}}{PP{h}_{i}} $, where *h*_*i*_ is the stutter peak height and *PPh*_*i*_ is the height of the parent allele peak (i.e., the height of the true peak that caused the stutter). The log likelihood for a sequence of stutter ratios (*r*_*i*_) is :

3 $$ {L_{r}=-{ \sum_{i = 1}^{n} \left({\log \left(v_{r}(x_{i}) \right) + { \frac { \left(r_{i} - u_{r}(x_{i}) \right)^{2}} {v_{r}(x_{i})^{2}}} } \right) }}  $$

If we define $ \left \{ \begin {array}{l} u(x_{i})=PP{h}_{i} \,.\,u_{r}(x_{i})\\ v(x_{i})=PP{h}_{i} \,.\,v_{r}(x_{i}) \end {array} \right. $, we see that the log likelihood for a sequence of stutter peak heights {*h*_1_,⋯,*h*_*n*_} is $ L_{h}=L_{r} - \sum _{i=1}^{n} \log \left (PP{h}_{i} \right) $, which is the log likelihood we use for comparing various stutter peak height models.

#### Drop-out

Drop-out events are denoted with binary indicator variables

$$y_{i}= {\huge \mathbbm{1}_{h_{i}}}=\left\{\begin{array}{l} 0 \;\text{if}\; h_{i} \geq 0,\\ 1 \;\text{if}\; h_{i}=0. \end{array} \right. $$

We model the probability of drop-out of an allele *i* with a function *p*(*x*)=*f*(*x*,*θ*) using decayed amplitude $x_{i}=A_{c}\ .\ e^{B_{c}. s_{i}}\phantom {\dot {i}\!}$, where *s*_*i*_ is the length of the allele *i* in base pairs. We estimate the set of parameters *θ* for the model using the Maximum Likelihood estimator *Θ*_*ML*_=arg max*θ*(*L*_*DO*_) over all *n* possible allele *i* with:

$$L_{DO} =-{\sum_{i=1}^{n} \log \left(\; p(x_{i}). y_{i} \; + \left(1 - p(x_{i}) \right) (1 - y_{i}) \;\right) } $$

### Estimation of the number of contributor (NOC) of a DNA sample

We extended the computation of *a posteriori* probability (APP) of the NOC *N*=*n* given a DNA sample (Evidence *E*) defined in [26] to account for differential (individual) degradation. To summarize the NOCIt algorithm developed in [26], the probability of observing evidence *E* (defined as the set of peaks in a DNA sample) given *N*=*n*,*P*(*E*|*N*=*n*), can be written as

$$P(E|N=n) \,=\, \sum_{\theta \in \mathcal{T}_{n}} P(E|N\,=\,n, \Theta \,=\, \theta) P(\Theta = \theta | N=n), $$

where *Θ* represents the fraction of the total sample from each contributor and each contributor’s degradation, and $\mathcal {T}_{n}$ is the set of all (discretized) possibilities of *Θ* compatible with *N*=*n*. Further, using the independence of genotypes across loci, we have

$$P(E|N=n, \Theta=\theta) = \prod_{l \in L} P(E_{l}|N=n,\Theta=\theta),$$

where *E*_*l*_ is the evidence at locus *l*.

At each locus *l*, NOCIt uses a Monte Carlo algorithm to generate random samples of *N*=*n* genotypes *g*_*l*_={*g*_*l*,1_,...,*g*_*l*,*n*_} and estimate *P*(*E*_*l*_,*G*=*g*_*l*_|*N*=*n*,*Θ*=*θ*). These estimates are used to calculate *P*(*E*_*l*_|*N*=*n*,*Θ*=*θ*) and, consequently, *P*(*E*|*N*=*n*). Finally, NOCIt calculates the APP according to

$$P(N=n | E)= \frac{P(E|N=n)}{\sum_{n=1}^{N_{max}}{P(E|N=n)}}.$$

## Supplementary information

**Additional file 1** Provides the optimum locus parameters values for four peak model components and 6 datasets.

**Additional file 2** Provides the optimum locus parameters values for four drop-out model components and 6 datasets.

## Data Availability

All data and material are publicly available at author’s portal : https://lftdi.camden.rutgers.edu/provedit/files/.

## Abbreviations

APP: A posteriori probability
CE: Capillary electrophoresis
DO: Drop-out
E: Evidence, set of observed peaks
L: Log likelihood
ML: Maximum likelihood
NP: Noise peak
NOC: Number of contributor
PCR: Polymerase chain reaction
PPH: Parent peak height
SP: Stutter peak
STR: Short tandem repeat
TP: True (Allelic) peak
